# Supplementary material for: A novel fracture lattice in spiny mouse skin facilitates tissue autotomy and regeneration
Source: bioRxiv. 2026 Mar 24:2026.03.23.713756. Preprint. [Version 1] doi: 10.64898/2026.03.23.713756 (PMC13042007; doi:10.64898/2026.03.23.713756)
Supplement: 1 [file NIHPP2026.03.23.713756V1-supplement-1.pdf]

bioRxiv preprint doi: https://doi.org/10.64898/2026.03.23.713756; this version posted March 24, 2026. The copyright holder for this preprint (which was not certified by peer review) is the author/funder, who has granted bioRxiv a license to display the preprint in perpetuity. It is made available under aCC-BY 4.0 International license.

Supplementary Video 1. Pinch load fracture tests in *Acomys* and *Mus* skin

Supplementary Video 2. Whole-mount staining of *Acomys* skin labeled by CNA35-EGFP

Supplementary Video 3. Pinch load simulation with and without *Acomys* skin pattern

Supplementary Video 4. Crack initiation simulation

Supplementary Video 5. Opening mode fracture tests in *Acomys* and *Mus* skin

Supplementary Video 6. Tearing mode fracture tests in *Acomys* and *Mus* skin

Supplementary Video 7. Crack propagation simulation in opening and tearing modes

bioRxiv preprint doi: https://doi.org/10.64898/2026.03.23.713756; this version posted March 24, 2026. The copyright holder for this preprint (which was not certified by peer review) is the author/funder, who has granted bioRxiv a license to display the preprint in perpetuity. It is made available under aCC-BY 4.0 International license.

Supplementary Notes

<u>Elastic moduli</u>

The elastic modulus is a fundamental material property that governs mechanical deformation, representing a material's resistance to deformation under applied stress. As illustrated in Fig. 2C, the skin of *Acomys* exhibits a unique structure, with collagen fibers encasing lipid clusters. To accurately predict the deformation behavior of *Acomys* skin, it is essential to measure the elastic moduli of both collagen and lipid. Since *Acomys* skin failure typically occurs under tensile loading, tensile experiments were conducted to characterize the tensile properties of the combined collagen-lipid system (Extended Data Fig. 4b). Additionally, atomic force microscopy (AFM)-based nanoindentation experiments were performed to determine the stiffness ratio between collagen and lipid (Extended Data Fig. 4c), allowing for the extraction of each material's modulus.

The elastic moduli of collagen and lipid used in the simulations were determined through volume averaging, based on tensile tests (Extended Data Fig. 4b) and nanoindentation results (Extended Data Fig. 4c). The volume-averaged elastic modulus $\bar{E}$ is defined as:

$$\bar{E} = \frac{E_{lip}V_{lip} + E_{col}V_{col}}{V_{lip} + V_{col}}, \qquad \text{(Eq. 1)}$$

where E and V represent the elastic modulus and volume, with the subscripts "lip" and "col" referring to lipid and collagen, respectively. Nanoindentation experiments revealed that the elastic moduli of collagen and lipid exhibit an approximate ratio of $E_{col}/E_{lip} = 10$ (Extended

bioRxiv preprint doi: https://doi.org/10.64898/2026.03.23.713756; this version posted March 24, 2026. The copyright holder for this preprint (which was not certified by peer review) is the author/funder, who has granted bioRxiv a license to display the preprint in perpetuity. It is made available under aCC-BY 4.0 International license.

Data Fig. 4c). Using this ratio and the volume-averaged elastic modulus $\bar{E} = 644$ kPa measured from tensile tests, the elastic moduli of collagen and lipid were calculated using Eq. 1, given their respective volumes.

To estimate the volume ratio, the structure of *Acomys* skin, depicted in Fig. 2C, was assumed, as illustrated in Extended Data Fig. 12a. Collagen clusters were modeled as parallelograms of length L, while the lipid width was defined as w. The unit cell of the lipid-collagen structure is shown in Extended Data Fig. 12b. Assuming $L = 200$ µm and $\theta = 60°$ for the lipid cluster and $w = 10$ µm for the collagen width, the volume ratio of lipid to collagen was calculated to be approximately 8.4. Substituting this ratio into Eq. 1, the elastic moduli of collagen and lipid were determined to be 3294 kPa and 329.4 kPa, respectively. These values were used in the finite element simulations. The Poisson's ratio was set to 0.475, consistent with values reported for mouse skin[57].

<u>Simulation under pinch loading without crack</u>

When *Acomys* is subjected to an attack from predators, out-of-plane loads (pinch loading) are initially applied to the skin. To examine the effects of the fracture lattice structure under such conditions, a model was developed, as shown in Extended Data Fig. 4a. Shell elements were employed, with the thickness set to 2 mm to match the actual skin thickness. Displacements and rotations in all directions were constrained on the four external surfaces of the structure. A force of 0.4 N was applied to the central cell of the structure in the out-of-plane direction. Two models were considered (Fig. 3a): the first model simulates a structure composed entirely of collagen in the absence of the fracture lattice structure with an elastic modulus of $E_{col} = 3294$ kPa. The second model represents a structure in which collagen surrounds lipid clusters, as depicted in

bioRxiv preprint doi: https://doi.org/10.64898/2026.03.23.713756; this version posted March 24, 2026. The copyright holder for this preprint (which was not certified by peer review) is the author/funder, who has granted bioRxiv a license to display the preprint in perpetuity. It is made available under aCC-BY 4.0 International license.

Fig. 3a. As shown in Fig. 3a, the presence of fracture lattice structure leads to significant stress concentration in the collagen fibers, increasing the likelihood of crack formation in the collagen.

Crack propagation under opening mode loading

Simulations were performed to investigate the influence of the fracture lattice structure on crack initiation under tensile loading. Crack propagation was modeled using contact debonding, which does not require an initial crack. In this approach, adhesive conditions are applied to the crack surface. The crack surface was defined using an oblique crack plane, as indicated by the dashed line in Extended Data Fig. 12c. The simulations were compared to assess the influence of the lipid structure on crack propagation in the presence of the fracture lattice structures (Extended Data Fig. 12d). Contact debonding models the crack surface as a contact interface, with springs placed between the crack faces (Extended Data Fig. 12e). The spring characteristics follow a traction-separation relationship (Extended Data Fig. 12f), where T^*, δ^*, and δ^c represent the material properties governing debonding. For this study, parameters measured from meat were used: $T^* = 13.4$ kPa and $\delta^c = 9.2$ mm[58].

Opening mode (Mode I) vs Tearing mode (Mode III)

Fracture modes are typically classified as opening mode (Mode I), shearing mode (Mode II), and tearing mode (Mode III). As discussed above, when out-of-plane forces are applied to the skin, tensile loading is expected to dominate. To investigate the force required for crack propagation in thin structures, simulations were performed for both opening and tearing modes (Fig. 1E). The simulation model was designed as shown in Extended Data Fig. 12g,h, aiming to minimize mode mixity and ensure the dominance of a single fracture mode. The specimen dimensions were set

bioRxiv preprint doi: https://doi.org/10.64898/2026.03.23.713756; this version posted March 24, 2026. The copyright holder for this preprint (which was not certified by peer review) is the author/funder, who has granted bioRxiv a license to display the preprint in perpetuity. It is made available under aCC-BY 4.0 International license.

as follows: total length of 20mm, thickness t of 2mm, and width w of 5mm. The initial crack length a was set to 2.5mm in the opening mode (Extended Data Fig. 12g) and 10mm in the tearing mode (Extended Data Fig. 12h) simulation, respectively. The crack propagation simulations were performed using an interface delamination approach, where crack propagation occurs when the energy release rate reaches a critical value. A linear fracture criterion was employed, assuming a critical energy release rate of 2 $\mu J/mm^2$ for all three fracture modes.

The crack propagation simulation videos are provided in Supplementary Video 7. The force-displacement curves derived from these simulations are presented in Fig. 4f, illustrating that crack propagation in opening mode requires substantially higher force. This observation aligns with predictions made by fracture mechanics theory. The sample geometry depicted in Extended Data Fig. 12g is commonly associated with fracture toughness predominantly governed by opening mode, and is referred to as a single edge notched tension (SENT) specimen. For this specimen, the fracture toughness can be analytically calculated as[59]:

$$K_I = \frac{P}{t\sqrt{w}} f\left(\frac{a}{w}\right), \qquad \text{(Eq. 2)}$$

where f is a function of the specimen geometry. In this study, the initial crack length-to-width ratio was set to 0.5, resulting in $f(a/w)$ of approximately 3.54[59]. Therefore, the energy release rate for the opening mode crack in Extended Data Fig. 12g is given by:

$$G_I = \frac{K_I^2}{E} = \frac{12.6P^2}{Et^2w}, \qquad \text{(Eq. 3)}$$

bioRxiv preprint doi: https://doi.org/10.64898/2026.03.23.713756; this version posted March 24, 2026. The copyright holder for this preprint (which was not certified by peer review) is the author/funder, who has granted bioRxiv a license to display the preprint in perpetuity. It is made available under aCC-BY 4.0 International license.

where E denotes the elastic modulus. For the calculation of the energy release rate in the tearing mode, the double cantilever beam model was employed. The energy release rate is given by:

$$G_{III} = \frac{\partial U}{\partial A} = \frac{24P^2a^2}{Et^4w}. \quad \text{(Eq. 4)}$$

Thus, when equal forces are applied in the opening and tearing directions of the crack, the energy release rate in the tearing mode is approximately $2(a/t)^2$ times higher than in the opening mode. In this study, with $a/t = 5$, the energy release rate in the tearing mode is approximately 50 times greater. Therefore, if the critical energy release rates for crack propagation are identical in both modes, cracks are more likely to propagate under tearing loading. However, for short crack lengths, G_I may exceed G_{III}. Consequently, when the crack length is less than $t/\sqrt{2}$, crack propagation occurs in the opening mode, whereas for longer cracks, propagation follows the tearing mode.

It is important to note that since the energy release rate for tearing mode was calculated using the double cantilever beam model in this section, this calculation may not be accurate for cases where the crack length-to-thickness ratio is small, as the Euler-Bernoulli beam assumption does not apply. Nevertheless, the fundamental insight that crack propagation initially occurs under opening mode, and subsequently transitions to tearing mode loading after some progression, remains valid.
